# Molecular Effect of Tobacco on Genetic, Epigenetic, and Metabolic Pathways During Cancer Progression

**DOI:** 10.7759/cureus.102757

**Published:** 2026-01-31

**Authors:** Ujjwal Kumar, G Jahnavi, Bijit Biswas, Benazir Alam, Saurabh Varshney

**Affiliations:** 1 Vital Strategies Tobacco Control Project, All India Institute of Medical Sciences, Deoghar, IND; 2 Community and Family Medicine, All India Institute of Medical Sciences, Deoghar, IND; 3 Otolaryngology, All India Institute of Medical Sciences, Rishikesh, IND

**Keywords:** carcinogenesis, dna damage, genetic alterations, nicotine, tobacco

## Abstract

Tobacco consumption remains a leading global health challenge, driving chronic diseases such as cancer, cardiovascular disorders, and metabolic dysfunction through intricate molecular mechanisms. This study investigates the multifaceted effects of tobacco exposure on genetic, epigenetic, and metabolic pathways, focusing on its role in carcinogenesis. Tobacco smoke, laden with carcinogens like benzopyrene, nitrosamines, and reactive oxygen species (ROS), induces genetic mutations and impairs DNA repair by downregulating tumor suppressor genes like tumor protein 53 (P53), ataxia-telangiectasia mutated (ATM), ataxia telangiectasia and Rad3-related (ATR), and poly (ADP-ribose) polymerase 1 (PARP1), leading to genomic instability and heightened cancer risk. Dysregulation of apoptosis-regulating genes B-cell lymphoma 2 (BCL-2), CL2 associated X (BAX), and cysteinyl aspartate specific proteinase 3 and 9 (CASPASE-3, CASPASE-9) further promotes tumor cell survival, while nicotine addiction genes cholinergic receptor nicotinic beta 3 subunit (CHRNB3), dopamine receptor D2 (DRD2), catechol-O-methyltransferase (COMT), and dopamine beta-hydroxylase (DBH) reinforce dependency via dopaminergic pathways. Metabolically, tobacco disrupts glycolysis, oxidative phosphorylation, and folate metabolism by altering cytochrome P450 family 2 subfamily A member 6 (CYP2A6), methylenetetrahydrofolate reductase (MTHFR), and hypoxia-inducible factor 1-alpha (HIF-1α) expression, resulting in insulin resistance, mitochondrial dysfunction, and lipid peroxidation, which exacerbate systemic diseases and cancer progression (Warburg effect). Epigenetic changes, including DNA methylation and histone modifications via histone deacetylase 1 (HDAC1), enhancer of zeste 2 polycomb repressive complex 2 subunits (EZH2), and suppressor of variegation 3-9 homolog 1 (SUV39H1), silence tumor suppressors cyclin-dependent kinase inhibitor 2A (CDKN2A), creating a long-term oncogenic imprint. Mitochondrial genes, mitochondrially encoded NADH: ubiquinone oxidoreductase core subunit 1 & 4 (MT-ND1 and MT-ND4) and mitochondrially encoded cytochrome c oxidase I (MT-CO1), suffer, reducing ATP synthesis and increasing ROS, which drives apoptosis evasion and inflammatory nuclear factor kappa B (NF-κB), interleukin 6 (IL-6), and tumor necrosis factor-α (TNF-α). This research uniquely integrates these molecular disruptions, emphasizing novel insights into metabolic reprogramming (CYP2A6, MTHFR, HIF-1α) and epigenetic mechanisms in tobacco-induced pathogenesis. Additionally, it explores impacts on stem cell genes, SRY-box transcription factor 2 (SOX2), octamer-binding transcription factor 4 (OCT4), and Nanog homeobox (NANOG), linking tobacco to cancer stem cell proliferation and metastasis (e.g., oral squamous cell carcinoma). The study also highlights tobacco’s role in aging, telomere shortening - telomerase reverse transcriptase (TERT downregulation) - and thymic involution, accelerating immunosenescence and disease susceptibility. These findings underscore the need for targeted interventions, such as epigenetic therapies, metabolic reprogramming, and robust tobacco control policies, to mitigate the global burden of tobacco-related diseases. By providing a unified framework for understanding tobacco’s molecular impact, this research advocates for precision medicine and public health strategies to address the pervasive effects of tobacco on human health.

## Introduction and background

Worldwide, tobacco addiction is the main cause of preventable disease. Every year, tobacco use directly causes the deaths of almost eight million individuals [1]. Globally, tobacco addiction is one of the biggest issues facing society on all fronts, social, political, industrial, economic, environmental, and medical. Tobacco grown on the Indian subcontinent is Nicotiana tabacum, a member of the Solanaceae family within the plant kingdom [2]. Tobacco leaves are processed into consumable produce that is smokeless, and for smoking, once crop harvesting is complete. Because nicotine is found in tobacco leaves and has a genuinely pleasurable impact, tobacco addiction is associated with activation of the dopaminergic system. Posselt and Raimann extracted and identified nicotine from Nicotiana tabacum for the first time in 1828. One class of alkaloids, namely 1-methyl-2-[3pyridyl]pyrrolidine, is nicotine. Many phytochemicals found in tobacco leaves are called alkaloids, and among these are at least 70 chemicals that are known to be carcinogenic (cancer-causing) and are found in tobacco smoke. These include nicotine, formaldehyde, hydrogen cyanide, arsenic, lead, ammonia, carbon monoxide, benzene, tobacco-specific nitrosamines (TSNAs), polycyclic aromatic hydrocarbons (PAHs), and radioactive substances like polonium-210. All of these compounds, including several that cause lung and heart problems, have been implicated in the development of cancer [3]. At the moment, tobacco addiction binds the entire human civilization. This article covers the fundamentals, the mechanism of addiction and its impact on genes and metabolism, the manner of misuse, the effects of tobacco, and the state of current approaches to a future free of tobacco.

This study aims to analyze the genetic and molecular alterations induced by tobacco exposure and their role in carcinogenesis. Specifically, it evaluates the impact of tobacco-related carcinogens on key tumor suppressor genes such as tumor protein 53 (P53), ataxia-telangiectasia mutated (ATM), ataxia telangiectasia and Rad3-related (ATR), and poly (ADP-ribose) polymerase 1 (PARP1), as well as apoptosis-regulating genes like B-cell lymphoma 2 (BCL-2), BCL2-associated X protein (BAX), and cysteinyl aspartate specific proteinase (CASPASE-3 and CASPASE-9), which are crucial for maintaining genomic stability and preventing tumor progression. Additionally, the study investigates how nicotine addiction modifies neurobiological and metabolic gene expression, reinforcing dependency while disrupting fundamental pathways such as glycolysis, oxidative phosphorylation, and folate metabolism. A critical aspect of this research is exploring the epigenetic modifications caused by tobacco exposure, including DNA methylation, histone modifications, and chromatin remodelling, which can lead to sustained oncogenic activation and genetic imprinting. Furthermore, the study examines the impact of tobacco-induced mitochondrial dysfunction on ATP synthesis, oxidative stress, and metabolic reprogramming, leading to cellular energy deficits and apoptosis evasion. It also evaluates how tobacco exposure activates inflammatory pathways, upregulating genes such as nuclear factor kappa B (NF-κB), interleukin 6 (IL-6), and tumor necrosis factor alpha (TNF-α), which sustain chronic inflammation, immune evasion, and tumor progression.

## Review

Mechanism of carcinogenesis by tobacco products

International Agencies of Research on Cancer (IARC) has classified over 2000 phenolic chemicals found in Nicotiana tabacum, the plant used to make most tobacco products, as carcinogenic. Of these, 55 have been found to be carcinogenic. This provided proof of easily established high carcinogenicity for animals and humans. Benzene, radioactive polonium, benzo[a]pyrene, N-nitrosamines, 4(Methylnitrosamino)-1-(3-pyridyl)-1-butanone (NNK), and other polycyclic hydrocarbons are the main carcinogens present in tobacco leaves [4]. The substance that is left in the glass filter is called tar, and the amount of harmful chemicals varies depending on the kind of tobacco product. For instance, hand-rolled cigarettes have more tar than filter cigarettes, and black (air-cured) tobacco has more TSNAs than blond flue-cured tobacco [5]. Smokers ingest 1-2 mg of nicotine every cigarette, which makes up 0.05-4% of the total weight of tobacco leaves [6]. The body quickly absorbed the extracted nicotine and it proceeded to enter various metabolic pathways, primarily forming (±80%) cotinine. By stimulating the brain's mesolimbic dopaminergic reward system, nicotine is the primary ingredient that causes habitual behavior, the continuation of tobacco use, and its addictive effects.

Several studies reported that increasing evidence established lung cancer as one of the most common carcinogens, which is caused by tobacco smoking sources like benzopyrene (B[a]P) or nitrosamine [4-(methylnitrosamine)-1-(3-pyridyl)-1-butanone NNK [7]. The malignancies for lung cancer by tobacco smoke due to benzopyrene have been well documented in various research with the A549 cell line. These studies show the role of benzopyrene, which promotes lung carcinoma in the A459 cell line and upregulates cytokine, interleukin 8 (IL-8) expression [7].

Nicotine has a double-ring structure containing carbon, hydrogen, and nitrogen (C10, H14, and N2). It is a colorless, volatile, strongly alkaline liquid in its pure state and turns pale yellow to dark brown. On exposure to air, it gives a characteristic tobacco smell [8]. After long-term extensive studies, it was found that nicotine has potentially lethal properties and that one drop of pure nicotine is sufficient to kill an animal or human within a few minutes [9]. In a case of chewable tobacco, nicotine is released due to the moisture of the tobacco leaf becoming its hydrophilic nature, while during smoking, nicotine volatilizes and mixes with smoke on minute droplets of tar as free nicotine. It not only acts as tolerance to its own action, like other addictive drugs [10]. Its consumption also triggered other drug addictions such as alcohol, Charas, and marijuana [11]. Through tobacco smoking, nicotine reaches the lungs and enters the bloodstream, whereas chewable tobacco’s nicotine enters the mucosal membrane of the mouth and nose or the skin. After pulmonary absorption, all types of chemicals present in smoke are circulated quickly into different parts of the body system [12]. After proper circulation into the body, nicotine rapidly reaches the brain within seven seconds [13,14]. This sudden supply of nicotine in the brain causes increasing blood pressure due to stimulation of the adrenal glands, resulting in the discharge of epinephrine. Due to this effect, there is also evacuation of the concentration of glucose, which increases respiration and heart rate. Nicotine also releases dopamine, therefore psychoactive rewards occur first, and these rewards are highly reinforced. Distribution of nicotine in the body generally takes place throughout but mostly to skeletal muscles and the brain that activates specific receptors, which are cholinergic receptors that influence cerebral metabolism. In the case of chewable tobacco, nicotine was able to quickly be absorbed from the lungs [13,15]. Chemically, nicotine is a structural analog to acetylcholine (Ach), where acetylcholine transfers information from one neuron to another in the form of electrical nerve impulses. Acetylcholine receptors in the body are most commonly recognized as nicotine receptors. It binds in the brain at nicotinic acetylcholine receptors (nAchRs) and influences the cerebral metabolism by stimulating these receptors. Nicotine acetylcholine receptors are triggered down impulses postsynaptic nerve fibers. Chronic addiction to nicotine inactivates and desensitizes nAChRs along with increases in the number of sites for nAChRs [16,17].

The effect of nicotine is also found in major psychiatric disorders by altering the neurotransmitters that dysfunction the pathogenesis of the leading psychiatric diseases, including norepinephrine, dopamine, serotonin (5-HT), gamma-aminobutyric acid (GABA), glutamate, and endogenous opioid peptides. Several radio diagnostic studies of tobacco addicted brains show that nicotine enhances the function and activation in the thalamus, prefrontal cortex, and visual system, consistent with activation of cortico-basal ganglia-thalamic brain circuits [18-21]. Therefore, it is the strongest reinforcing agent in both animals and humans. Cholinergic receptors are present in the midbrain area, such as the mid-brain tegmental in which accumbens (Nas), nucleus, striatum, and the ventral tegmental area [16]. All these receptors are activated by acetylcholine. Besides binding to nAChRs, nicotine also binds to cholinergic receptors in the autonomic ganglia and adrenal medulla. The cholinergic receptors are relatively large structures having many components known as subunits. nAChRs are composed of 12 subunits in mammalian brain, 9 alpha (α) subunits (α2 to α10) and 3β subunits (β2 to β4), which play the central role in autonomic transmission [22].

Many electrophysiological studies revealed that nicotine agonists triggered the release of GABA from the rodent brain, and release is Ca2+ dependent [23]. The actions of nicotine on ventral tegmental GABAergic innervation, which modulates the mesolimbic dopamine excitability [24]. Whereas, acute nicotine administration stimulates the release of noradrenaline (NA) in the different parts of the brain, primarily at the locus coeruleus level [25]. This decreases the concentration of 5-HT in the hippocampus [26].

Genetics and epigenetics effect of nicotine

**Figure 1:**
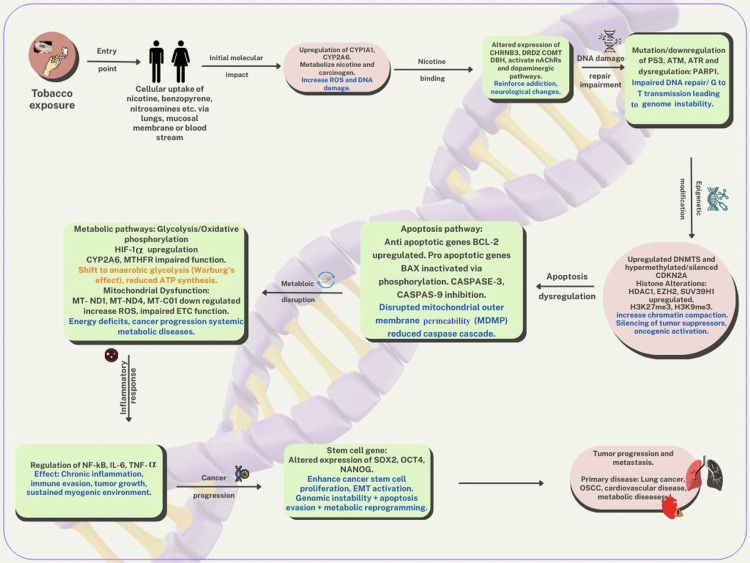
Schematic diagram illustrating the molecular impact of nicotine on gene expression in cancer progression. Nicotine disrupts genes (PARP1, P53, PTEN, BCL-2, BAX, CASPASE-3/9, SOX2, OCT4, NANOG), increasing ROS, genomic instability, and cancer stem cell proliferation, promoting tumor initiation and metastasis. ATM, ataxia-telangiectasia mutated; ATR, ataxia telangiectasia and Rad3-related; ATP, adenosine triphosphate; BAX, BCL2-associated X; BCL-2, B-cell lymphoma 2; CASPASE-3/9, cysteinyl aspartate-specific proteinase 3 and 9; CDKN2A, cyclin-dependent kinase inhibitor 2A; CHRNB3, cholinergic receptor nicotinic beta-3 subunit; COMT, catechol-O-methyltransferase; CYP2A6, cytochrome P450 family 2 subfamily A member 6; DBH, dopamine beta-hydroxylase; DNMTs, DNA methyltransferases; DRD2, dopamine receptor D2; ETC, electron transport chain; G, guanine; H3K27me3, histone 3 lysine 27 trimethylation; H3K9me3, histone 3 lysine 9 trimethylation; HIF-1α, hypoxia-inducible factor-1 alpha; IL-6, interleukin-6; MT-ND1, mitochondrially encoded NADH:ubiquinone oxidoreductase core subunit 1; MT-ND4, mitochondrially encoded NADH:ubiquinone oxidoreductase core subunit 4; MTHFR, methylenetetrahydrofolate reductase; NANOG, Nanog homeobox; NF-κB, nuclear factor kappa B; OCT4, octamer-binding transcription factor 4; OSCC, oral squamous cell carcinoma; PARP1, poly(ADP-ribose) polymerase 1; P53, tumor protein 53; ROS, reactive oxygen species; SOX2, SRY-box transcription factor 2; T, thymine; TNF-α, tumor necrosis factor alpha. Original schematic illustration created by the authors with data from [22,27-30] using Canva

It is clear that nicotine acts as an addictive neurotransmitter that is responsible for dependency on tobacco-caused cancer. But many individuals have different responses to smoking and tobacco addiction. Every addiction begins with the first cigarette, which is no exception. Many first-time tobacco smokers show a range of responses, such as dizziness and nausea. Various phenotypic responses can be attributed to genetic variation in the neuronal cholinergic receptor nicotinic gene (CHRN). This is a subunit gene of the nAChR receptor that absorbs nicotine and activates the receptors (as shown in Figure 1), initiating a cascade of downstream neurobiological effects [27]. Many studies suggest that CHRN genes are involved in the reward pathway in addictive behaviors. Several CHRN genes are associated with tobacco-related phenotypes [28]. Many studies based on gene expression of candidate gene CHRNB3 gene have variation in the putative promoter region that is associated with nicotine dependence [29]. During early experimentation with cigarettes and chewing tobacco, the gene CHRNB3/A6 emerged as the strangest signal early subjective response that triggers ‘dizziness” in the initial startup of smoking or tobacco chewing. The mechanism of dazing is a single-nucleotide polymorphism (SNP) on CHRNB3 gene due to nicotine binding [30].

Numerous genetic studies [31] have demonstrated a substantial genetic contribution to many aspects of tobacco addiction, as summarized in Figure 1. Genetic variations also regulate nicotine pharmacokinetics such as continine metabolized in almost 75% of nicotine, whereas continine itself is metabolized to trans-3′-hydroxy-cotinine. This reaction is primarily catalyzed by cytochrome P450 family 2 subfamily A member 6 (CYP2A6) and liver cytochrome P450 (CYPs) enzyme [32]. The CYP enzymes are the constituents of the group of six CYP genes, which are highly polymorphic, with the most common wild type allele CYP2A61 and other polymorphisms have been identified, including duplication, deletion, insertion, and single nucleotide polymorphism (s). Several studies have failed to explain the exact functional activity of these polymorphisms, but it is clear that the SNPs reduce enzymatic metabolism, in which CYP2A6 has a maximum metabolic capacity [33].

The psychoactive effect of nicotine directly stimulates cognitive functions like learning, attention and memory through nAChR activation. nAChR excitation provokes burst firing in subcortical dopamine-secreting neurons that are part of the mesocorticolimbic dopamine pathway [34]. The dynamic increase in dopamine is due to the reinforcing effect of nicotine. Generally, all addictive drugs activate dopaminergic circuitry [35]. In addition, dopamine is also released by GABAergic, glutamatergic, and opioidergic mechanisms. The genetic variation and functional differences in these circuits show that nAChRs are composed of 5α and β subunit combinations. All these subunits, α4β2, have high potential for nicotine. However, many studies reported that α4 subunits of nAChRs are sufficient for nicotine tolerance, reinforcement and sensitization [36]. On the other hand, dopamine is broken down by two enzymes that are dopamine β-hydroxylase and catechol-O-methyltransferase, which are encoded by dopamine beta-hydroxylase (DBH) and catechol-O-methyltransferase (COMT) gene, respectively [37]. Polymorphism of these genes that encode the dopamine receptor subtype has been associated with smoking and the motivation status of cigarettes. Some significant investigations show that a gene that also affects central dopaminergic function like polymorphism is the 3′ untranslated region of dopamine receptor D2 (DRD2) gene, which is approximately twice as common in smokers compared with non-smokers [38]. This association has been linked to the Taq1 restriction fragment length polymorphism (Taq1 RFLP), which arises from a single nucleotide substitution (C→T) at position 32806 within the dopamine receptor D2 (DRD2/ANKK1) gene locus and has been correlated with increased susceptibility to smoking behavior. This analysis correlates the polymorphism with smokers [39]. Basically, it was established that CYP2D6 is responsible for hyperoxylation of nicotine to continine and the CYP2D6 phenotype associated with smoking addiction, but this has not been validated by molecular typing [32]. However, a recent experimental study suggested that CYP2D6 does not play a major role in nicotine metabolism, but studies explained that CYP2A6 play a more important role than CYP2D6 in nicotine metabolism due to the minor differences in amino acid sequences at the substrate recognition site between CYP2D6 and CYP2A6. This is the reason behind the fastest nicotine oxidation by CYP2A6 [33]. It is concluded that the main biosynthetic pathway for dopamine is from tyrosine through tyrosine hydroxylase, whereas CYP2D6 also converts tyramine, which is a major dietary amine to dopamine [33]. This alternative pathway is physiologically important but affected by genetically determined variation in the CYP2D6 pathway. These genetically determined variations in cytochrome activity might also be important in determining the response to pharmacological therapy for tobacco dependence.

Nicotine affecting gene expression in cancer progression

The amount of nicotine used by tobacco addicts ultimately caused cancer and, in this section, we discuss what and how nicotine affects the carcinoma associated genes like p53 apoptosis effector related to PMP-22 (PERP), B-cell lymphoma 2 (BCL-2), Bcl-2-associated X protein (BAX), cysteine-dependent aspartate-directed protease (CASPASE-3 and CASPASE-9), suppressor of cytokine signaling 3 (SOCS-3), phosphatase and tensin homolog (PTEN), tumor protein 53 (P53), ataxia-telangiectasia mutated (ATM), ataxia telangiectasia and Rad3-related (ATR), SRY-box transcription factor (SOX), epithelial cell adhesion molecule (Ep-CHEM), cytokeratin 19 (CK-19), octamer-binding transcription factor (OCT), and homeobox transcription factor Nanog (Nanog).

PARP Gene

P53 apoptosis effector related to peripheral myelin protein 22 (PMP22) is a plasma membrane protein that, in humans, is encoded by the PERP gene. PERP is a direct transcriptional target of p53, but its transcription can also be regulated by other transcription factors. Poly (ADP-ribose) polymerase1 transferase, which modifies various nuclear proteins by poly(ADP-ribosyl)ation. The modification is dependent on DNA and is involved in the regulation of various important cellular processes such as differentiation, proliferation, and tumor transformation and also in the regulation of the molecular events involved in the recovery of cells from DNA damage. A detailed study hypothesized that the role of the P21-PARP1 axis is regulating cigarette smoking caused DNA damage that induces lung cancer. Some evidence also shows that cigarette smoking increases the level of P21 protein in lung microphages and epithelial cell lines 547 and lungs [40], in which poly (ADP-ribose) polymerase1 (PARP-1), and NAD+ - dependent DP-ribosyltransferase1 are reported as damage sensors, which bind to damage DNA and initiate the cellular response to DNA single-strand breaks and double-strand breaks [41].

BCL-2 Gene

Serine threonine-protein phosphate (BCL-2 gene) acts as a pro-survival factor and controlling membrane permeability of mitochondria. BCL-2 also regulates mitochondria apoptotic factors like cytochrome C or BAX to cytosol; due to this, it suppresses the programmed cell death process. A patient with carcinoma of the lung, head and neck has upregulated expression of BCL-2 due to heavy exposure to tobacco smoke [42].

BAX

Bax also belongs to a main proapoptotic member of the TL BCL-2 family. This is essential for apoptotic cell death. BAX is abundantly expressed in both small cell lung cancer and non-small cell lung cancer, and growth factor-induced phosphorylation of BAX has been reported to negatively regulate its proapoptotic function. Studies reported that nicotine promotes survival of human lung cancer cells in a novel mechanism by inactivating the proapoptotic function of BAX via its phosphorylation [42,43].

Nicotine contains 0.6-3.0% of the dry weight of tobacco, which is absorbed by the lungs and transported in blood stream [13,25]. The half-life of nicotine is 1-2 hours. It is lipid soluble thereby highly permeable to the plasma membrane and reaches the intracellular space. The nicotine metabolism takes place in the liver, and its emission takes place in the kidneys [44]. Nicotine metabolism inhibits the production of antioxidant enzymes, therefore increasing lipid peroxidation and this reaction leads to the production of free radicals (reactive oxygen species (ROS)). These free radicals reach tissues and initiate damaging of cytoplasmic membrane and DNA fragments. The increasing free radicals create multiple causes for alteration in mitochondrial apoptotic pathways. Pre-apoptotic genes such as Bax and Bid and anti-apoptotic genes like BCL-2 and BCL-XL are all BCL-2 family participatory proteins engaged in regulating balance between cell life and cell death. Association of BSL-2 and Box prevents mitochondrial oligomerization and translocation, which stimulates permeability transition and activates Caspase, which triggers Caspase 9 and induces the activation of Caspase cascade 9 [45]. Activation of the intrinsic apoptotic pathway involves mitochondrial outer membrane permeabilization (MOMP), which results in the release of cytochrome c into the cytosol. This event promotes apoptosome formation and activation of initiator caspases such as Caspase-9. Activated Caspase-9 subsequently cleaves and activates executioner caspases, including Caspase-3, leading to programmed cell death. Nicotine exposure disrupts this cascade by impairing caspase activation and attenuating p53-mediated apoptotic signaling [46].

P53

P53 is a housekeeping and tumor suppressor gene that controls the cell cycle and protects cells from DNA damaging against biological and environmental risk factors including cigarette smoking. In the development of carcinogenesis in human cells, p53 becomes inactive due to altering MDM2 or P19ARF gene; therefore, p53 common aberration in human cancer [47]. A detailed study has been experimented in correlation between gene expression of p53/p21 and tobacco-smoking patients of esophageal squamous carcinoma. Investigate the immunohistochemistry expression of p53 and p21 in 80 patients with esophageal squamous cell carcinoma in connection to potential risk factors, such as tobacco use, and determine whether their expression is associated with a better prognosis in terms of p53-dependent and -independent pathways - cancer stem cells (CSCs).

PTAN Gene

Although direct experimental evidence linking nicotine exposure to PTEN suppression remains limited, emerging studies suggest that tobacco smoke-associated signaling pathways may indirectly influence PTEN activity through dysregulation of the PI3K/AKT pathway. Given PTEN’s central role as a tumor suppressor regulating cell proliferation and survival, such indirect modulation may contribute to tobacco-associated carcinogenesis. PTEN (phosphatase and tensin homolog) is a crucial tumor suppressor gene that plays a significant role in regulating cell growth, proliferation, and survival [48]. It acts as a negative regulator of the PI3K/AKT signalling pathway, which is involved in cell survival and proliferation [49]. Loss or mutation of PTEN can lead to uncontrolled cell growth and contribute to the development of various types of cancer, including breast, prostate, endometrial, and brain tumors [50]. Interestingly, while not directly related to nicotine, some studies have shown that certain substances can affect PTEN expression and activity. For example, caffeine has been found to induce PTEN activation and Akt inactivation, leading to the prevention of tumor cell proliferation in osteosarcoma and fibrosarcoma cells [51]. This suggests that external factors can potentially modulate PTEN activity and impact cancer cell growth. In conclusion, while the specific effects of nicotine on the PTEN gene are not addressed in the provided context, understanding the role of PTEN in cancer development and its potential modulation by external factors could be relevant for future research into the impact of substances like nicotine on tumor suppressor genes. Table 1 shows all missing genes included and specific immune cells mentioned.

**Table 1:** List of nicotine-induced alterations in gene expression contributing to various pathological conditions, disorders, and diseases.

Gene Name	Expression Site	Normal Function	Dysfunction After Nicotine Exposure	Leading Disease	References
Poly (ADP-ribose) polymerase 1 (PARP1)	Nucleus	DNA repair, cell survival	Dysregulated DNA repair, increased DNA damage	Lung cancer	[52]
B-cell lymphoma 2 (BCL-2)	Mitochondria	Anti-apoptotic, regulates mitochondrial membrane permeability	Overexpression, suppression of apoptosis	Lung, head, and neck cancers	[53]
BCL2-associated X protein (BAX)	Mitochondria	Pro-apoptotic, regulates programmed cell death	Inactivated via phosphorylation, reduced apoptosis	Lung cancer	[54]
Cysteinyl aspartate specific proteinase 3 (CASPASE-3)	Cytoplasm	Apoptosis execution	Inhibition of caspase cascade, impaired apoptosis	Various cancers	[55]
Cysteinyl aspartate specific proteinase (CASPASE-9)	Cytoplasm	Initiates apoptosis cascade	Dysregulated mitochondrial apoptosis	Cancer progression	[56]
Suppressor of cytokine signaling 3 (SOCS-3)	Macrophages, T-cells, B-cells	Cytokine signaling regulation	Overexpression leading to immune suppression	Lung cancer, inflammation-related diseases	[57]
Phosphatase and tensin homolog (PTEN)	Cytoplasm, nucleus	Tumor suppression, cell cycle regulation	Downregulated, enhanced PI3K/AKT signaling	Prostate, breast, endometrial cancer	[58,59]
Tumor protein 53 (P53)	Nucleus	Tumor suppression, DNA damage response	Mutations, loss of function	Lung, esophageal, head, and neck cancer	[60,61,62]
Ataxia-telangiectasia mutated (ATM)	Nucleus	DNA damage repair	Reduced expression, impaired DNA repair	Genomic instability, cancer	[63]
Ataxia telangiectasia and Rad3-related (ATR)	Nucleus	DNA replication stress response	Impaired function, increased mutations	Cancer progression	[64]
SRY-box transcription factor 2 (SOX2)	Neural stem cells, embryonic stem cells	Regulates self-renewal and differentiation	Overexpression in carcinomas	Oral, lung cancer	[65]
SRY-box transcription factor 2 (SOX3)	Thymus, hematopoietic stem cells, B-cells, T-cells, macrophages	Regulates early neural development and immune cell differentiation	Overexpression in immune cells, leading to immune suppression and tumorigenesis	Hematological malignancies, immune dysfunction	[66]
Octamer-binding transcription factor 4 (OCT4)	Embryonic, cancer stem cells	Regulates pluripotency	Increased expression in tumors	Oral squamous cell carcinoma	[67]
Nanog homeobox (NANOG)	Embryonic, cancer stem cells	Maintains stem cell self-renewal	Upregulated, leading to cancer stem cell proliferation	Lung, oral cancer	[68]
Epithelial cell adhesion molecule (Ep-CAM)	Epithelial cells	Cell adhesion, signaling	Increased expression, promotes metastasis	Carcinomas	[69]
Cytokeratin 19 (CK-19)	Epithelial tissues	Cytoskeletal organization	Upregulated in malignancies	Carcinomas	[70]
Methylenetetrahydrofolate reductase (MTHFR)	Liver, kidney, blood cells	Folate metabolism, DNA synthesis	Impaired function, increased homocysteine	Cardiovascular disease, cancer	[71]
Cytochrome P450 family 2 subfamily A member 6 (CYP2A6)	Liver	Nicotine metabolism	Genetic polymorphisms affecting nicotine clearance	Nicotine dependence, lung cancer	[33,72]
Dopamine beta-hydroxylase (DBH)	Brain, adrenal medulla	Dopamine metabolism	Altered dopamine processing, affecting addiction	Tobacco dependence	[73]
Catechol-O-methyltransferase (COMT)	Brain, liver	Dopamine and catecholamine breakdown	Affects dopamine levels, increases smoking motivation	Nicotine addiction	[74]
Dopamine receptor D2 (DRD2)	Brain	Dopamine receptor function	Polymorphisms linked to smoking behavior	Nicotine addiction	
Mitochondrially encoded NADH: ubiquinone oxidoreductase core subunit (MT-ND1 and MT-ND4), mitochondrially encoded cytochrome c oxidase I (MT-CO1)	Mitochondria	Oxidative phosphorylation	Impaired ATP synthesis, increased ROS	Neurodegenerative disorders, cardiovascular diseases	[75]
Sirtuin 1 (SIRT1)	Nucleus, cytoplasm	Chromatin regulation, DNA repair	Suppressed, leading to reduced cell longevity	Aging, metabolic disorders	[76]
Cyclin-dependent kinase inhibitor 2A, also known as P16INK4a (CDKN2A)	Nucleus	Tumor suppression, cell cycle control	Hypermethylation, silencing	Cancer, aging	[77]
Forkhead box N1 (FOXN1)	Thymus epithelial cells	Thymus development, T-cell maturation	Downregulated, impaired immunity	Immunosenescence, increased infection risk	[78]
Hypoxia-inducible factor 1-alpha (HIF-1α)	Nucleus	Hypoxia response	Upregulated, promoting tumor survival	Lung, pancreatic cancer	[79]
Interleukin 6 (IL-6)	Immune cells (macrophages, T-cells)	Inflammation, immune response	Overexpression leading to chronic inflammation	Autoimmune diseases, cancer	[80]
Tumor necrosis factor alpha TNF-α	Macrophages, monocytes	Inflammation, apoptosis regulation	Increased expression, tissue damage	Chronic inflammation, cancer	[81]
Nuclear factor kappa B (NF-κB)	Immune cells, epithelial cells	Regulates inflammation and immune response	Chronic activation, leading to tumor progression	Inflammatory diseases, cancer	[82]
Heat shock protein 90 (HSP90)	Cytoplasm	Protein folding, stress response	Overexpression in response to tobacco, promotes cancer cell survival	Lung, head, and neck cancers	[83]
Telomerase reverse transcriptase (TERT)	Nucleus	Telomere maintenance	Downregulated, leading to accelerated aging	Cancer, premature aging	[84]
DNA polymerase gamma (POLG)	Mitochondria	mtDNA replication and repair	Dysfunction leading to mitochondrial mutations	Aging, neurodegeneration	[85]

Metabolic disruptions caused by tobacco

Tobacco and nicotine exposure have profound impacts on multiple metabolic pathways, significantly altering glycolysis, the Krebs cycle, and folic acid metabolism, alongside lipid metabolism, glucose homeostasis, and oxidative stress response [86]. Nicotine, a major bioactive component in tobacco, interferes with energy metabolism by affecting glycolysis and mitochondrial oxidative phosphorylation. Nicotine and tobacco carcinogens such as PAHs and nitrosamines disrupt glycolytic enzymes like hexokinase and pyruvate kinase, reducing ATP production and shifting cellular metabolism towards anaerobic glycolysis, a phenomenon resembling the Warburg effect observed in cancer cells. Furthermore, the Krebs cycle (tricarboxylic acid cycle, TCA) is significantly impaired due to increased ROS production, which inactivates key enzymes such as α-ketoglutarate dehydrogenase and succinate dehydrogenase, leading to mitochondrial dysfunction and metabolic reprogramming [87,88]. The inhibition of AMP-activated protein kinase (AMPK) and sirtuins by nicotine further dysregulates energy homeostasis, promoting insulin resistance and fat accumulation. In addition, tobacco exposure alters folic acid metabolism by reducing folate absorption and increasing homocysteine levels, leading to DNA hypomethylation and genomic instability. The downregulation of enzymes like methylenetetrahydrofolate reductase (MTHFR) and thymidylate synthase impairs nucleotide synthesis, increasing susceptibility to DNA damage and mutagenesis [89]. Epigenetic modifications, including DNA methylation and histone acetylation, further alter gene expression in metabolic pathways, affecting genes such as peroxisome proliferator-activated receptor gamma (PPAR-γ), fat mass and obesity associated (FTO), and insulin receptor substrate 1 (IRS1), which regulate adipogenesis and glucose metabolism. These disruptions collectively contribute to metabolic disorders, including insulin resistance, dyslipidemia, and cardiovascular diseases, by promoting systemic inflammation through NF-κB activation and endothelial dysfunction [90,91]. Table 2 summarizes the major metabolic pathways affected by nicotine, their normal functions, dysfunction after nicotine exposure, and the resulting diseases.

**Table 2:** List of major metabolic pathways disrupted by nicotine exposure.

Metabolic Pathway	Normal Function	Dysfunction After Nicotine Exposure	Leading Disease	Reference
Glycolysis	Converts glucose into pyruvate for ATP production	Shift towards anaerobic glycolysis (Warburg effect), reduced ATP production	Cancer progression, insulin resistance	[92]
Krebs cycle (TCA cycle)	Generates ATP, NADH, and flavin adenine dinucleotide reduced (FADH2) for oxidative phosphorylation	Disrupted enzyme activity (e.g., α-ketoglutarate dehydrogenase, succinate dehydrogenase), increased ROS	Mitochondrial dysfunction, metabolic disorders	[93]
Oxidative phosphorylation (electron transport chain, ETC)	Produces ATP via mitochondrial respiration	Increased ROS, impaired Complex I-IV function, cytochrome C release	Neurodegenerative diseases, cardiovascular disorders	[94]
Folate metabolism	DNA synthesis, repair, and methylation	Reduced folate absorption, increased homocysteine, DNA hypomethylation	Cancer, cardiovascular diseases, birth defects	[95]
Lipid metabolism	Breakdown and synthesis of lipids for energy and membrane formation	Increased lipid peroxidation, dyslipidemia, fatty liver	Cardiovascular diseases, fatty liver disease	[96]
Glucose homeostasis (insulin signaling pathway)	Regulates blood glucose levels via insulin signaling	Increased insulin resistance, impaired AMP-activated protein kinase (AMPK) function	Type 2 diabetes, obesity	[97]
Glutathione metabolism (antioxidant defence)	Neutralizes oxidative stress and maintains redox balance	Reduced glutathione levels, increased oxidative stress	Cancer, neurodegeneration, chronic inflammation	[98]
Catecholamine metabolism (dopamine, epinephrine, norepinephrine pathway)	Regulates mood, stress response, and addiction	Altered dopamine signalling, increased addiction potential	Nicotine addiction, mood disorders	[99,100]

Tobacco effect on stem cell genes

Oct-4, Sox and Nanog are transcription factors that play an important role in necessary gene expression of self-renewal embryonic and adult stem cells. Apart from all other transcription factors, Sox2, Oct4 and Nanog also play a major role in the maintenance of stem cells [101] from approximately 1600 transcription factors encoding gene in human genome [102]. The SOX family of transcription factors comprises approximately 20 members, classified into several subgroups based on the presence of a conserved SRY-related high-mobility group (HMG) DNA-binding domain [103]. These proteins are categorized from SOXA to SOXH according to structural similarity within the HMG domain [104]. SRY is the sole member of the SOXA group, while the SOXB family is subdivided into SOXB1 (SOX1, SOX2, SOX3) and SOXB2 subgroups. Members of the SOXB1 subgroup, particularly SOX2, function cooperatively with OCT4 by binding shared DNA motifs to regulate genes essential for pluripotency and self-renewal. It creates a synergistic effect using formation of heterodimers in regulation of their target gene [67,105,106]. Nanog is another important transcription factor that is only expressed in undifferentiated cells. These three play a significant role in stem cell regeneration, and during cancer development, alteration in their expression is observed [107]. In tobacco-associated carcinogenesis, particularly oral squamous cell carcinoma, dysregulation of stem cell-associated transcription factors such as SOX2, OCT4, and NANOG has been observed across tumor initiation, progression, and metastatic stages. These alterations contribute to the maintenance and expansion of epithelial-derived cancer stem cell populations, supporting tumor aggressiveness and therapeutic resistance [107,108].

Smoking signature: G (guanosine) to T (thymine) transversion

Scientists have discovered that the DNA-carcinogen adducts preferentially form at guanine bases by observing them at the atomic scale. In the DNA helix, guanine bases typically couple with cytosine bases. However, the enzymes responsible for replicating DNA with an adduct often place an adenine base, rather than the typical cytosine, opposite this guanine when the DNA is copied. As a result, a G-to-T transversion occurs [109]. Different mutations are brought about by different environmental conditions. For instance, CC to TT (cytosine-cytosine to thymine-thymine) mutations are produced by ultraviolet light. These noticeable alterations might make it easier to determine which treatments are best for a certain individual [110].

Smokers' lung cancers are more likely to include p53 mutations than non-smokers' lung cancers. A significant proportion of p53 mutations seen in lung malignancies are G→T transversions, a subtype of mutation uncommon in tumors other than hepatocellular carcinoma. Prior research has demonstrated a strong association between regions of preferential production of PAH adducts along the p53 gene and GT transversion hotspots in lung malignancies [111]. It has been noticed that p53 mutations in lung cancer are different from those in other cancers and that an excess of G→T transversions is characteristic of these tumors [112]. G→T transversions have been likened to a ‘molecular signature’ of tobacco smoke mutagens in smoking-associated lung cancers for the following reasons: (i) PAHs are prominent carcinogens in tobacco smoke that can produce predominantly this type of mutation [113]; (ii) PAH adducts are present in DNA extracted from human tissues exposed to tobacco smoke [114]; (iii) there is an increased frequency of G→T transversions in lung cancers from smokers compared with lung cancers from non- smokers and compared with most other cancers [115]; (iv) a non-transcribed strand bias of G→T transversions can be attributed to preferential repair of adducts on the transcribed strand [113]; and (v) there is a precise correspondence between mutational hotspots and hotspots of adduct formation by PAHs found in tobacco smoke [116].

Epigenetic modifications and histone alterations

Tobacco exposure induces widespread epigenetic modifications, significantly altering gene expression through DNA methylation, histone modifications, and chromatin remodeling. Nicotine and tobacco-derived carcinogens disrupt normal histone protein function by modifying histone acetylation, methylation, and phosphorylation, leading to aberrant gene regulation. These modifications affect transcription factors, DNA repair mechanisms, and cellular homeostasis, contributing to oncogenesis, aging, and metabolic dysfunction [117-119].

Histone acetylation is crucial for gene activation, as it relaxes chromatin structure and facilitates transcription factor binding. Nicotine exposure disrupts this process by increasing the activity of histone deacetylases (HDACs) such as HDAC1, HDAC2, and HDAC3, which remove acetyl groups from histones [120,121], leading to chromatin compaction and transcriptional repression. The hyperactivity of HDACs results in the silencing of tumor suppressor genes like cyclin-dependent kinase inhibitor 2A (CDKN2A and P16INK4a), promoting uncontrolled cell proliferation. Additionally, tobacco exposure downregulates HAT (histone acetyltransferases), including p300 / CREB-binding protein (p300/CBP), further limiting histone acetylation and reducing gene activation necessary for DNA repair and cellular longevity [122].

Histone methylation also plays a pivotal role in gene expression regulation, and its dysregulation due to nicotine exposure contributes to cancer progression and cellular aging. Tobacco carcinogens upregulate EZH2 (Enhancer of Zeste Homolog 2), a key component of the Polycomb Repressive Complex 2 (PRC2), which catalyses histone 3 lysine 27 trimethylation (H3K27) trimethylation (H3K27me3). This modification represses transcription of tumor suppressor genes such as PTEN and cadherin 1 CDH1, impairing apoptosis and enhancing metastasis. Simultaneously, nicotine downregulates lysine demethylase 6A (KDM6A) and ubiquitously transcribed tetratricopeptide repeat on X chromosome (UTX), a histone demethylase responsible for removing H3K27me3 marks, leading to sustained repression of these tumor suppressors [123].

Tobacco exposure also affects histone phosphorylation, a modification essential for DNA damage response and chromatin remodelling. The phosphorylation of histone 2AX (H2AX) and gamma histone 2AX (γH2AX) is a critical marker of DNA double-strand breaks, and nicotine-induced oxidative stress increases γH2AX levels, indicating heightened DNA damage [124]. However, tobacco exposure simultaneously downregulates ATM (ataxia telangiectasia mutated) and ATR (ataxia telangiectasia and Rad3-related protein), two key kinases involved in DNA damage repair. This dysregulation impairs the recruitment of DNA repair proteins, promoting genomic instability and increasing the likelihood of malignant transformation [125].

The effects of histone modifications extend to transcription factor regulation, further influencing gene expression patterns. Nicotine suppresses the expression of p53, a master regulator of apoptosis and cell cycle arrest, by enhancing the recruitment of EZH2 and HDAC1 to its promoter region, leading to H3K27me3 and histone deacetylation [126]. This repression inhibits p53-dependent tumor suppression, allowing damaged cells to evade apoptosis and accumulate mutations. Similarly, the NF-κB pathway, which regulates inflammation and immune responses, is dysregulated by tobacco exposure. Nicotine-induced HDAC3 activation leads to histone deacetylation at NF-κB target genes, suppressing immune surveillance and promoting chronic inflammation [127].

Mitochondrial dysfunction and ATP production disruption

Tobacco exposure severely impairs mitochondrial bioenergetics by inducing oxidative stress, mitochondrial DNA (mtDNA) damage, and dysfunctional electron transport chain components. The detrimental effects of tobacco on mitochondrial function are primarily mediated by ROS and reactive nitrogen species present in tobacco smoke, which disrupt mitochondrial homeostasis [128,129].

Nicotine and other tobacco-derived carcinogens interfere with Complex I nicotinamide adenine dinucleotide reduced (NADH: ubiquinone oxidoreductase), the primary entry point of electrons into the electron transport chain (ETC). Chronic exposure to nicotine causes excessive production of ROS, leading to oxidative modification of Complex I subunits, impairing electron transfer efficiency, and resulting in electron leakage [130]. This leakage exacerbates oxidative damage by further generating superoxide radicals, which subsequently convert into highly reactive hydroxyl radicals, accelerating mitochondrial dysfunction. Additionally, nicotine upregulates pro-apoptotic proteins such as BAX and downregulates anti-apoptotic proteins like BCL-2, facilitating cytochrome c release from mitochondria, thereby triggering caspase activation and apoptotic cell death [131].

Complex II (succinate dehydrogenase) is also negatively impacted by tobacco exposure. Tobacco-induced mitochondrial stress leads to succinate accumulation, contributing to pseudo-hypoxic conditions that enhance oncogenic signaling through the stabilization of hypoxia-inducible factor 1-alpha (HIF-1α). This phenomenon promotes metabolic reprogramming, shifting energy production from oxidative phosphorylation to aerobic glycolysis (Warburg effect), a hallmark of cancer metabolism that facilitates rapid cellular proliferation despite reduced ATP yield [132].

Tobacco-induced oxidative stress further disrupts Complex III (cytochrome BC1 complex), exacerbating electron leakage and ROS generation [133]. The inhibition of Complex III function results in an imbalance of mitochondrial membrane potential, leading to defective proton pumping and ATP synthesis. ROS-mediated oxidative damage to cardiolipin, a phospholipid essential for maintaining Complex III stability, results in impaired supercomplex formation and reduced ETC efficiency. Similarly, Complex IV (cytochrome c oxidase), responsible for the final transfer of electrons to oxygen, is also targeted by tobacco carcinogens, which impair its enzymatic activity and decrease ATP synthesis, further compounding mitochondrial dysfunction [134].

The impairment of ATP synthase (Complex V) due to nicotine exposure leads to a reduction in ATP generation, affecting cellular energy metabolism. Tobacco smoke alters the proton gradient across the inner mitochondrial membrane, disrupting ATP synthesis and promoting mitochondrial permeability transition pore (mPTP) opening, which facilitates the release of pro-apoptotic factors into the cytoplasm. This cascade of events culminates in mitochondrial-mediated apoptosis, a key factor in smoking-induced tissue degeneration and organ dysfunction [135].

Apart from direct effects on ETC complexes, tobacco exposure contributes to mitochondrial DNA (mtDNA) damage. Unlike nuclear DNA, mtDNA lacks protective histones and robust repair mechanisms, making it highly susceptible to oxidative damage. Tobacco smoke-derived ROS induce mtDNA mutations and deletions, impairing mitochondrial gene expression and leading to the synthesis of defective ETC proteins [136]. Mutations in mtDNA contribute to mitochondrial heteroplasmy, a condition where functional and non-functional mitochondria coexist, further exacerbating energy production deficits [137].

Tobacco-induced mitochondrial dysfunction is also linked to endoplasmic reticulum (ER) stress and autophagic dysregulation. Nicotine alters calcium homeostasis, leading to ER stress-mediated mitochondrial calcium overload, which further disrupts ATP production and triggers apoptosis [138]. Additionally, tobacco components impair mitophagy, the selective degradation of damaged mitochondria via the autophagy pathway. The downregulation of key mitophagy regulators, such as (PTEN induced kinase 1) PINK1 and parkin RBR E3 ubiquitin protein ligase (Parkin), leads to the accumulation of defective mitochondria, increasing ROS production and cellular damage [139].

Smoking, aging, and telomerase shortening

The aging process is fundamentally influenced by tobacco consumption through oxidative stress and telomere attrition. Tobacco smoke generates an excess of ROS, which damage cellular components, leading to premature senescence. ROS interact with cellular macromolecules, causing lipid peroxidation, protein misfolding, and DNA strand breaks [140]. Telomeres, the repetitive DNA sequences at the ends of chromosomes, progressively shorten with each cell division [141]. Their maintenance by telomerase, primarily through TERT (telomerase reverse transcriptase), is crucial for cellular longevity [142]. However, tobacco exposure significantly downregulates TERT, reducing telomerase activity and accelerating telomere shortening, leading to genomic instability and increased cellular aging. The reduction in telomerase activity results in increased apoptosis, higher susceptibility to age-related diseases such as cardiovascular disorders, neurodegeneration, and cancer [143]. Additionally, nicotine-induced chronic inflammation exacerbates these effects by increasing pro-inflammatory cytokines such as IL-6, TNF-α, and NF-κB, which further contribute to oxidative stress and systemic aging [144].

The inflammatory cascade triggered by tobacco exposure further accelerates cellular aging. Chronic exposure upregulates NF-κB, a key transcription factor involved in inflammatory responses, which promotes the release of IL-6 and TNF-α. These inflammatory mediators contribute to oxidative stress, immune system dysregulation, and persistent cellular damage. Elevated levels of P16INK4a (CDKN2A), a tumor suppressor and key marker of aging [145,146], are induced by tobacco exposure. Increased P16INK4a expression inhibits cell cycle progression, forcing cells into an irreversible state of growth arrest. This phenomenon is commonly observed in aged tissues and is particularly pronounced in the lungs of long-term smokers, where cellular repair mechanisms are severely compromised.

Tobacco/nicotine effects and the thymus theory of aging

The thymus, a primary lymphoid organ essential for the development and maturation of T-cells, plays a critical role in maintaining adaptive immunity [147]. However, with advancing age, the thymus undergoes progressive involution, leading to a decline in T-cell production and a weakened immune response. This process, often referred to as immunosenescence, predisposes individuals to infections, autoimmune disorders, and malignancies [148,149]. The thymus theory of aging [150] posits that the deterioration of thymic function is a major contributor to age-associated immune dysfunction. Smoking and chronic nicotine exposure further accelerate thymic involution through oxidative stress, inflammation, and disruption of key intracellular signalling pathways involved in thymocyte differentiation. Nicotine exerts profound effects on thymocyte development by interacting with nicotinic acetylcholine receptors (nAChRs) expressed on thymic epithelial cells (TECs) and thymocytes. These receptors play a critical role in intracellular signalling, and their dysregulation by chronic nicotine exposure alters T-cell selection and differentiation processes. Nicotine binding to nAChRs disrupts calcium-dependent signalling pathways, leading to increased apoptosis of developing thymocytes and reduced T-cell output [151].

One of the primary mechanisms by which smoking induces thymic atrophy is through oxidative stress. Tobacco smoke contains ROS and other free radicals that cause direct damage to TECs and thymocytes. Excessive ROS generation activates nuclear factor kappa B (NF-κB), a transcription factor that regulates the expression of inflammatory cytokines such as interleukin-6 (IL-6), tumor necrosis factor-alpha (TNF-α), and interleukin-1 beta (IL-1β) [152]. These pro-inflammatory cytokines create an inhospitable microenvironment in the thymus, promoting TEC apoptosis and impairing thymocyte maturation. Furthermore, oxidative stress disrupts the expression of key genes involved in thymic homeostasis. Forkhead box N1 (FOXN1), a transcription factor essential for TEC development and function, is significantly downregulated in smokers. This reduction in FOXN1 expression leads to a decline in TEC proliferation and thymic epithelial integrity, accelerating thymic involution. Additionally, the upregulation of p53, a tumor suppressor gene associated with apoptosis and cellular senescence, further contributes to thymocyte loss [153].

Beyond direct effects on the thymus, smoking-induced stress responses exacerbate immune dysfunction. Chronic tobacco use leads to elevated systemic levels of cortisol, a glucocorticoid hormone known to suppress thymic function [154]. High cortisol concentrations induce apoptosis in thymocytes and inhibit TEC proliferation, further exacerbating thymic atrophy. Additionally, smoking increases the secretion of catecholamines (epinephrine and norepinephrine), which impair thymocyte migration and differentiation via beta-adrenergic receptor activation. As a result of these molecular and cellular changes, smokers exhibit profound immune deficits. The reduced output of naive T-cells compromises immune surveillance, increasing susceptibility to viral and bacterial infections. Moreover, the altered T lymphocytes (T-cell) repertoire contributes to the pathogenesis of autoimmune diseases by disrupting self-tolerance mechanisms [155].

Common screening tests for tobacco-related cancers

Tobacco’s role in driving cancers through genetic, epigenetic, and metabolic disruptions, such as P53 mutations, DNA methylation, and the Warburg effect, underscores the need for early detection to halt disease progression. For lung cancer, linked to carcinogens like benzo[a]pyrene and nitrosamines, low-dose computed tomography (LDCT) is recommended for high-risk smokers (aged 50-80 with a 20 pack-year history), detecting small nodules for early-stage treatment via surgical resection, potentially achieving remission in non-small cell lung cancer [152,153]. Sputum cytology and chest X-rays, though less sensitive, may complement LDCT. OSCC, driven by tobacco-induced oxidative stress and stem cell gene overexpression (SOX2, OCT4, NANOG), is screened through oral visual examinations to identify precancerous lesions like leukoplakia [154], with toluidine blue staining, brush biopsy/cytology, and fluorescence imaging aiding detection. Early OSCC can be treated with surgical excision or laser therapy, preventing invasive cancer. Head and neck cancers, associated with BCL-2 upregulation, are screened via physical examinations, endoscopy (laryngoscopy/pharyngoscopy), biopsies, and CT/MRI imaging [155], enabling curative surgery or radiation for early-stage cases. Esophageal cancer, tied to P53 and P21 dysregulation, is detected using upper endoscopy (EGD), barium swallow X-rays, or chromoendoscopy, with early interventions like endoscopic resection preventing progression. Pancreatic cancer, fuelled by nitrosamines and HIF-1α, is screened in high-risk individuals with endoscopic ultrasound (EUS), CT/MRI, or CA 19-9 blood tests, though early detection is rare, and surgical resection offers a chance for cure. Bladder cancer, linked to CYP1A1 induction, is screened with urine cytology, cystoscopy, urine biomarker tests (e.g., nuclear matrix protein 22 - NMP22), and CT urography, allowing treatments like transurethral resection of bladder tumor (TURBT) for non-muscle-invasive cancers [156], often leading to remission. Smokers, especially those with genetic predispositions (e.g., CYP2A6 polymorphisms), are prime candidates for these screenings. While lung, oral, and bladder cancer screenings are well-established, pancreatic cancer lacks routine protocols due to cost and incidence concerns. Integrating these tests with cessation programs, as advocated by the document, can reduce cancer incidence and improve outcomes, supporting precision medicine and public health strategies.

## Conclusions

This study highlights the profound impact of nicotine and tobacco on genetic expression, metabolic pathways, and cellular function, driving chronic diseases, especially cancer. Nicotine, a highly addictive compound, reinforces neurobiological mechanisms while modulating oncogenes, tumor suppressors, and regulatory pathways. Dysregulation of apoptosis, DNA repair, and immune responses accelerates carcinogenesis, systemic inflammation, and metabolic disorders. Disruption of genes such as p53, BAX, BCL-2, CASPASE-3, and PTEN, alongside alterations in glycolysis, oxidative phosphorylation, and folate metabolism, illustrates the molecular underpinnings of tobacco-related pathology. Nicotine-induced epigenetic modifications, including DNA methylation and histone changes, establish long-term genetic imprinting, emphasizing the need for targeted interventions like epigenetic modifiers, antioxidant therapy, and precision medicine. Beyond molecular insights, this research underscores urgent tobacco control strategies-cessation programs, advertising bans, taxation, and early genetic screening for high-risk groups. Bridging molecular biology, public health, and clinical interventions, it advances precision medicine while reinforcing multidisciplinary action against tobacco’s global health burden.
